# Treatment of Impetigo

**Published:** 1874-11

**Authors:** 


Treatment of Impetigo—*By Dr. Wiltshire.*—"No disease of the skin yields more gratifying results than this. The first thing to be aimed at is the improvement of the general health; and as the patients are usually the subjects of scrofulosis, and not unfrequently rickety, this is the best accomplished by improving the diet, by change of air, and by the exhibition of cod-liver oil and steel-wine. Quinine is also extremely useful in this disease. It can readily be added to vinum ferri. Now and then the syrup of iodide of iron suits well, and especially if there be otorrhœa. Attention should also be paid to the bowels.

"Locally, as a rule, it is best not to attempt to remove the crusts by poulticing, etc. They will soon come off under appropriate treatment, and no time is lost or pain caused by attempts at their premature removal. The application of an ointment consisting of ten, fifteen or twenty grains of white precipitate to an ounce of zinc ointment is attended by brilliant results. The effect of this ointment is very striking. It should be applied twice or even thrice a day. The scalp should always be searched and the hair cut off from and around all suspicious spots.

"The skin may be cleansed by oat or almond meal and water, made of the consistence of thin cream without boiling. No danger need be apprehended from the white precipitate. In many hundreds of cases I have never seen any evil results or sign of absorption follow; on the contrary, its use has been attended by the most gratifying results. This preparation of mercury is not readily absorbed from the skin, and besides, its use is rarely required for more than a very short time. Occasionally, and especially in the presence of pediculi and their nits, the use of an ointment composed of equal parts of white precipitate und sulphur ointment is preferable and answers well."—*The American Practitioner.*

